# Effect of Microwave Treatment on Protease Activity, Dough Properties and Protein Quality in Sprouted Wheat

**DOI:** 10.3390/foods13081277

**Published:** 2024-04-22

**Authors:** Xiangyu Wang, Mengyuan Zhao, Panpan Shang, Jing Liu, Renyong Zhao

**Affiliations:** 1 National Engineering Research Center of Wheat and Corn Further Processing, Henan University of Technology, Zhengzhou 450001, China; 2 College of Food Science and Technology, Henan University of Technology, Zhengzhou 450001, China; 3 Food Laboratory of Zhongyuan, Luohe 462300, China

**Keywords:** microwave treatment, sprouted wheat, protease inactivation, dough properties, gluten quality, cross-linking

## Abstract

In this study, the effects of microwave treatment on protease activity, dough properties and protein quality in sprouted wheat were investigated. Microwave treatment led to a significant (*p* < 0.05) reduction in protease activity in sprouted wheat. Proteases with a pH optimum of 4.4 (cysteine proteinases) were more susceptible to microwave heating, which contributed mostly to protease inactivation. Significant improvements (*p* < 0.05) in the dough properties and gluten quality of sprouted wheat were observed, which are probably attributable to the synergistic effectiveness of protease inactivation and heat-induced gluten cross-linking. After microwave treatment, the decrease in the solubility and extractability of protein in sprouted wheat indicated protein polymerization, which was induced by intermolecular disulfide bond cross-linking. The changes in gliadin were less pronounced due to the relatively low temperature of the microwave treatment. The cross-linking in sprouted wheat that occurred after microwave treatment seemed to mainly involve glutenin, especially B/C low-molecular-weight glutenin subunits (B/C-LMW-GSs) in the range of 30–50 kD.

## 1. Introduction

Pre-harvest sprouting of wheat results in the production of a range of hydrolytic enzymes, such as α-amylase, proteases and endoxylanase [1], which cause deleterious effects on the quality of end-use products. Typically, α-amylase is regarded as the predominant enzyme of endogenous origin in sprouted wheat, and it has been extensively studied [1,2,3,4]. In the past, great efforts aiming to minimize sprouting damage mostly focused on inactivation of α-amylase. Traditional heating treatments, including steaming [5], dry-air heating [6] and extrusion [7], were applied to improve the quality of sprouted wheat by inactivating the hydrolytic enzymes. But these conventional heating methods were time-consuming with low energy effectiveness because, in these heating systems, heat was transferred by conduction from the kernel surfaces to the inner endosperms, while the heat conductivity of the wheat kernels was as low as that of the insulating materials [8]. Microwave heating, an energy-efficient technique, is an alternative to traditional heating and has gained in popularity for a wide variety of food and agricultural products due to its advantage of faster heating, energy efficiency and selective heating [9]. Microwave heating has been evidenced to reduce α-amylase activity and improve the quality of sprouted wheat [8,10]. Nevertheless, little has been known about the effect of microwave treatment on protease and protein in sprouted wheat. 

Protein, especially gluten, is primarily responsible for the unique viscoelastic and gas-retaining properties of dough made from wheat [11]. The proteolytic enzymes in wheat and their role in affecting the quality of dough by the alteration of gluten proteins have been the subject of considerable research. During wheat sprouting, the proteolytic activity increases strikingly due to the synthesis and secretion of endopeptidases into the starchy endosperm, germ and aleurone layer [12]. Protease rapidly hydrolyzes the insoluble storage proteins into small molecular compounds, peptides and amino acids, leading to rapid softening and weakening of wheat gluten [13,14]. Consequently, doughs prepared from sprouted wheat exhibit lower water absorption, decreased development time, higher stickiness and insufficient elasticity [15]. Furthermore, the adverse effects of sprouting on wheat-based products, such as bread [16], cakes [3], noodles [17] and pasta [18], have been extensively studied. Undesirable product characteristics, for example, inferior crumb, coarse texture, poor gas retention and low volume, are observed in end-use products made from sprouted wheat flour and are generally unacceptable to producers and consumers.

Several classes of proteinases have been detected in dry and germinating wheat. While serine proteases, metalloproteinases and aspartic proteinases are present in dry seeds, cysteine proteinases appear after the onset of germination [19]. They are defined by their different hydrolytic pH optima, including neutral and acidic endo-proteases. The majority of acidic proteases are cysteine proteinases (approximately 90%) [19,20], which are involved in the initial mobilization of storage proteins and participate in their further hydrolysis. Cysteine is extremely important for the structure and functionality of gluten because most cysteines form either intrachain disulfide bonds within proteins or interchain disulfide bonds between proteins [11]. Hydrolysis of cysteine by cysteine proteinases in sprouted wheat possibly leads to the cleavage of disulfide bonds in protein and thus protein depolymerization [21]. It was previously reported that gluten softening was caused by a reduction in the disulfide cross-links which develop during sprouting as a result of protease activity [22]. Capocchi [23] also found that cysteine proteinase, which was purified from wheat seeds at the fourth day of germination, was extremely effective in producing remarkable protein degradation in a short time and thus a loss of elasticity in the polymeric network of gluten. In a previous study, cysteine protease was reported to be stable up to 30 °C and to lose 45% of its activity at 50 °C [24]. The excessive proteolytic activity in suni-bug-damaged wheat can be inactivated by means of thermal treatment and steam tempering at about 70 °C for 1–5 min. In addition, the protease inactivation induced by microwave treatment in suni-bug-damaged wheat was observed by Diraman [25], who found that proteolytic activity decreased with microwave treatment time. However, the effects of microwave treatment on proteases in sprouted wheat, especially the cysteine proteinases, have not yet been comprehensively investigated.

In addition, heating treatment resulted in significant changes in the function and conformation of proteins, especially gluten. Baking [26], steaming [27] and extrusion [28,29] steps during cereal-based food production induced the reshuffling of exiting and the formation of additional intermolecular disulfide (SS) cross-linking, thereby forming a strong gluten network. Heating treatment resulted in a remarkable reduction in protein solubility and extractability by increasing the protein molecular weight [30]. As is well known, wheat endosperm storage proteins consist of two major groups of proteins, monomeric gliadins and polymeric glutenins [11]. They are extremely important and develop into gluten upon mixing flour with water and are of great technological importance in many food systems. In previous studies, it was generally accepted that glutenin was more susceptible to heat treatment than gliadin. When it was heated above 55 °C, and when gliadins were heated above 70 °C, disulfide/sulfhydryl (SH) exchange reactions occurred [29]. At very high temperature conditions (up to 90 °C), gliadin links to glutenin through a sulfhydryl–disulfide exchange mechanism [31,32]. The effect and mechanism of heating treatment on the physico-chemical properties and cross-linking of gluten have been extensively studied [33,34,35,36]. Furthermore, studies have reported changes in the wet gluten content of wheat after microwave drying [8], and the functionality and conformation of gluten altered gradually with an increase in exposure to microwave drying [37,38]. Although a number of studies have clarified the heat-induced changes that occur in gluten, there has been no systematic and in-depth study on gluten cross-linking in sprouted wheat after microwave treatment, especially considering protease inactivation.

In this study, the effects of microwave treatment on protease inactivation, dough properties and gluten cross-linking in sprouted wheat were investigated, aiming to elucidate improvement mechanisms for sprouted wheat after microwave treatment.

## 2. Experimental Section

### 2.1. Preparation of Sprouted Wheat Samples

Two wheat cultivars (ZM366 and TK6#) were obtained from the Henan academy of wheat breeding research center. The protein contents (%) of TK6# and ZM366 were 13.85 ± 0.05 and 14.94 ± 0.02, respectively. In order to standardize the samples, the sprouting of wheat was performed in a laboratory according to Singh [39] with some modifications. Wheat kernels were soaked in water at a temperature 4 ± 1 °C for 12 h and then washed with water. The excess water was drained off, and the superficial water present on the wheat kernels was removed. The soaked samples were sprouted for 8, 10, 12, 16, 20, 24 and 48 h at 20 °C in a temperature-controlled incubator. Sprouted wheat kernels were obtained after being carefully pre-dried in a convection air dryer at 40 °C for 8 h to a final moisture content of 12–14%.

### 2.2. Microwave Treatment of Sprouted Wheat Kernels

Tempering was performed to obtain the gradient distribution of moisture in the sprouted wheat kernels before microwave treatment. The sprouted wheat kernels were tempered for 45 min to a moisture content of 15.5%, and the amount of tempering water was calculated according to the initial moisture content of the sprouted wheat. After tempering, the sprouted wheat kernels (500 g in each experiment) were subjected to microwave treatment at a power level of 700 W in a microwave system (ER-761MD, Qingdao Haier, Qingdao, China) with a magnetron of 2450 MHz and an inner space volume of 40 L. All samples were milled at the automatic laboratory flourmill LRMM8040-3-D. The protein content (N × 5.7) and gluten index of ZM366 flour were 13.71% and 92%, while those of TK6# flour were 12.13% and 55%. The milled samples were sealed in polyethylene bags for further use. The microwave treatment times were 0, 30, 60, 90,120, 150 and 180 s. And there were at least two batches for each experiment.

### 2.3. Extraction of Protease

In sprouted wheat, the proteolysis enzymes consisted of two major types of protease with optimum pH levels of 7.5 and 4.4. In order to measure protease activity in the different pH conditions, wholewheat flour (2.0 g) was stirred for 2 h with 10 mL of sodium phosphate buffer (50 mM, pH 7.5) [40] and 10 mL of sodium acetate buffer (0.2 M, pH 4.4) [12]. The suspension was centrifuged at 3000× *g* for 10 min, and the supernatant (protease extraction) was used for the analysis of protease.

### 2.4. Measurement of Protease Activity

After pre-incubation for 2 min at 40.0 ± 0.2 °C, the protease extraction of 2 mL was reacted with 2 mL preheated tyrosine solution for 10 min in a test tube. Then, the reaction was terminated by the addition of 4 mL of 0.4 mol/L trichloroacetic acid (TCA) [40]. After centrifugation at 1000× *g* for 10 min, the absorbance of the supernatant was measured at 275 nm against the blank. A control was prepared by adding TCA prior to the tyrosine. Absorbance values were calibrated against the absorbance of *L-tyrosine* at 275 nm and converted to level of protease activity (μ/g).

The calculation for activity was as follows:

protease activity(ug)=∆A×K×82×110×n×E

$$∆A=A\left(reaction\right)-A(blank)$$

where *K* is the light-absorption coefficient; 8 is the total volume of the reaction mixture (mL); 2 is the volume of protease extraction (mL); 1/10: 10 is the reaction time (min), calculated with 1 min; *n* is the dilution ratio; and *E* is the conversion coefficient (0.5) between the method of ultraviolet absorption and Folin.

The calculated activity is given by the following equation:

protease activity(ug)=25×∆A×K×n×E

### 2.5. Measurement of Dough Tensile Properties

The extensograph test was performed according to AACC (2000) [41]. Dough for the extensograph test was prepared in a Brabender Farinograph^®^-E with an addition of 6 g of sodium chloride dissolved in one part water. Approximately 300 g of wheat flour (corrected to 14% moisture basis) was mixed in a 300 g mixing bowl until a 500 BU consistency was reached. The dough was stretched using a Brabender Extensograph^®^ (Brabender OHG, Duisburg, Germany) until rupture after resting for 45, 90 and 135 min in a humidified chamber (>90% relative humidity) conditioned at 30 °C.

### 2.6. Determination of Dough Mixing Properties and Gluten Quality

Mixing and pasting behaviors of wheat flour dough were measured using the Mixolab (Chopin, Tripette et Renaud, Paris, France), which is capable of evaluating the rheological properties of wheat flour. It measured in real time the torque (expressed in Nm) produced by passage of the dough between the two kneading arms, thus allowing the study of the physico-chemical behavior of the dough and the quality analysis of the protein network. For the assays, the appropriate amounts of flour and water (calculated automatically by Mixolab software (Version 3.20) according to the input values for water absorption and moisture in dry flour) were placed into the Mixolab analyzer bowl and subjected to hydration, mixing and heating according to the standard “Chopin+” protocol. The settings used in the test were 8 min at 30 °C, with a temperature increase of 4 °C/min until the mixture reached 90 °C, followed by a temperature decrease of 4 °C/min until the mixture reached 55 °C and then 6 min of holding at 55 °C. 

The quantity and quality of wet gluten were determined by washing gluten from wheat meal using an automatic washer, the Glutomatic 2200 (Perten Instruments, Stockholm, Sweden), according to Approved Method 38-12 (AACC international 2000) [41]. After being washed from the flour meal, the gluten was put in a special sieve and centrifuged under standard conditions. Depending on the rheological properties, a part of the gluten passed through the meshes of the sieve. The part of gluten that did not pass through the sieve was expressed as the percentage of total washed gluten and was defined as the gluten index (GI) [8].

### 2.7. Determination of Protein Solubility

Flour proteins of sprouted wheat were fractionated into salt-soluble (0.5 M sodium chloride), alcohol-soluble (70% ethanol) and acetic acid (0.05 M)-soluble and -insoluble fractions according to the modified Osborne fractionation method [42]. The nitrogen contents of each fraction in sprouted wheat flour after microwave treatment were determined using the Kjeldahl method. Nitrogen values were multiplied by 5.7 to convert them to protein values.

### 2.8. Size-Exclusion High-Performance Liquid Chromatography (SE-HPLC)

Sprouted wheat flour (1.00 g) was mixed with 20 mL 50% 1-propanol aqueous [43] by continuous vortexing for 1 min in a 50 mL centrifuge tube to disperse the flour. The protein extraction was performed in a constant temperature oscillation incubator by continuous shaking for 2 h. The suspension was centrifuged at 3000 r/min for 10 min and filtrated with 0.45 μm millex. For the purpose of increasing the extractability of insoluble polymeric protein, the ultrasound technique was applied before continuous shaking [44]. The slurry was sonicated with a Scientz-IID sonic dismembrator at an output of 95 W for 6 min. Samples were sonicated automatically at 1 sec intervals during extraction. To prevent heat buildup, sample tubes were placed in an ice bath during sonication.

The protein extracts of 20 μL were loaded on a Biosep-SEC-S4000 column with a separation range from 15 to 500 kDa (300 mm × 7.8 mm, Phenomenex, Torrance, CA, USA), and a Waters HPLC system with automated injection was used. The elution solvent was acetonitrile/water (1:1, *v*/*v*) containing 0.05% (*v*/*v*) trifluoroacetic acid (TFA), with a flow rate of 1.0 mL/min [35] and a column temperature of 40 °C. Proteins were detected by measuring UV absorbance at 214 nm. The elution profiles were divided into two fractions using the lowest absorbance reading between the two peaks as the cutoff point. The first peak (a retention time of 9~14 min) consisted of glutenin polymers, whereas the second peak (14~20 min) consisted mainly of gliadin monomers. Total extractable gluten, glutenin and gliadin were calculated from the corresponding peak areas.

### 2.9. Sodium Dodecyl Sulfate–Polyacrylamide Gel Electrophoresis (SDS-PAGE) Analysis

SDS-PAGE analysis of proteins was performed in a Bio-Rad vertical electrophoresis system (Protean II xi Cell, Bio-Rad Laboratories, HERCULES, Hercules, CA, USA) using 10% separating gel (pH 8.8) and 5% stacking gel (pH 6.8). The extraction buffer for protein was 62.5 mmol/L Tris-HCl (pH 6.8), containing 2% (*w*/*v*) SDS, 10% (*v*/*v*) glycerol and 0.1% (*w*/*v*) bromphenol blue. Wheat samples were extracted with 0.5 mL of 0.5 mol/L sodium chloride by continual shaking for 2 h. After centrifugation at 10,000 r/min for 10 min, the supernatant was mixed with the extraction buffer at a ratio of 1:1 (*v*/*v*). After mixing with deionized water twice to remove the albumin/globulin fraction, the residue was extracted with 0.5 mL of 70% (*v*/*v*) ethanol by continual shaking for 2 h and the supernatant was dried in an oven at 40 °C to obtain the gliadin fraction. Subsequently, the gliadin fraction was stirred with 0.3 mL of extraction buffer containing 5% β-mercaptoethanol (β-ME). The residue was then shaken with 0.5 mL of extraction buffer (containing 5% β-ME) for 12 h, yielding the glutenin fraction. The electrophoresis was conducted at a voltage of 95 V till the indicator reached the top of the separating gel and then increased to 110 V for the remainder of the run. Then, the gel was stained with 0.08% (*w*/*v*) Coomassie Brilliant Blue G-250 and de-stained in 10% acetic acid.

### 2.10. Reversed-Phase High-Performance Liquid Chromatography (RP-HPLC) Analysis

Protein extraction and RP-HPLC were conducted according to a previous study [34]. Wheat flour (100 mg) was mixed twice with 1 mL 50% 1-propanol by continuous vortexing in a 50 mL centrifuge tube to disperse the flour and centrifuged at 10,000 r/min for 10 min. The supernatants from each extract were pooled 1:1 and then filtered with 0.22 μm millex, yielding the gliadin fraction. The residue was then extracted with 1 mL 50% 1-propanol (containing 2 mol/L urea, 0.2 mol/L Tris-HCl (pH 6.6) and 1% (*w*/*v*) dithiothreitol (DTT)) by continuous shaking at 65 °C for 1 h. After centrifugation, 10 μL of 4-vinylpyridine was added to 0.8 mL of the supernatant to promote alkylation, avoiding repolymerization of the reduced subunits, and the mixture was continuously shaken at 65 °C for 30 min. After cooling, the mixture was filtered and separated by an XBridge BEH300 C18 column (5 μm, 4.6 mm × 250 mm, Waters, Milford, MA, USA). The elution system consisted of water (solvent A) and acetonitrile (solvent B), both containing 0.1% (*v*/*v*) trifluoroacetic acid (TFA). The flow rate was 1 mL/min, with a column temperature maintained at 60 °C.

The gliadin fraction was separated with gradient elution, in which solvent B increased from 25 to 50% over 45 min, followed by a drop from 50 to 25% in 9 min, and it was then held for 15 min. For the glutenin fraction, the solvent B was held at 24% for 7 min, then increased from 24 to 31% in 16 min, followed by increases from 31 to 32% in 6 min and from 31 to 48% over 38 min and a subsequent decrease from 48 to 24% over 5 min, and, finally, it was held for 3 min. 

### 2.11. Statistical Analysis

All the data obtained in the study are expressed as the means of at least two replicate determinations. Results are presented as mean values ± standard deviations based on dry matter. Non-parametric testing was applicated, and at least two replicas were used. Analysis of variance was performed using the software SPSS 2016 (SPSS Inc., Chicago, IL, USA).

## 3. Results and Discussion

### 3.1. Effect of Microwave Treatment on Protease Activity in Sprouted Wheat

Two types of protease have been found in germinated wheat, and they are defined by their different hydrolytic pH optima. As shown in Table 1, an initial small increase in protease activity after sprouting for 12 h was detected, followed by a 2- to 4-fold rise after 24–48 h of germination, ranging from 3.78 to 35.91 cu/g at pH 7.5 and from 190.89 to 535.51 cu/g at pH 4.4. These endo-proteases showed higher activity at pH 4.4 than at pH 7.5. Therefore, it could be inferred that most of the endo-proteases in the sprouted wheat kernels were acidic. The major enzyme, the protease with a pH optimum of 4.4, accounted for more than 90% (calculated from Table 1) of the overall proteolytic activity in sprouted wheat, showing a predominant role of the acidic protease (pH 4.4) in both wheat varieties. This was consistent with previous reports [23,45], which showed that acidic protease activity largely increased upon germination due to enzyme synthesis. Fahmy [24] reported that cysteine protease had an optimum pH of 4.0 in sodium acetate buffer. Gänzle [46] also summarized that cysteine proteinases were regarded as the most active protease group in germinated grains and generally showed highest activity in gluten hydrolysis at pH 4. It could be concluded that the acidic proteases (pH 4.4) of sprouted wheat in this study might have been cysteine proteinases and that the bulk of proteolytic activity (approximately 90%) during wheat germination was attributable to them [47].

In our pre-experiment, longer times for the microwave treatment, for example, 210 s, caused the wheat kernels to have a slightly burnt taste. Therefore, a microwave treatment time of 150 s was selected. A significant reduction in protease activity (Table 1) was observed in both types of proteases after microwave treatment for 150 s. Protease activity at pH 7.5 in TK6# decreased to less than 50% of the original activity after microwave treatment, and the decrease ratio remained relatively constant over the sprouting time. At an early stage of sprouting, the protease activity at the pH optimum of 4.4 significantly decreased after microwave heating, contributing mostly to the loss of total protease activity. This indicated that the acidic protease was more susceptible to microwave heating than the neutral one. Nevertheless, with the increase in sprouting time, the decrease ratio of protease activity at pH 4.4 in TK6# decreased from 86.3 to 12.07%. A similar trend was also observed in ZM366. According to a previous study [12], the acidic protease (pH 4.4) was found mainly in the outer layers of wheat kernels, while the neutral protease (pH 7.5) was endosperm-specific. In theory, more microwave energy was absorbed by the outer layers of the wheat kernels due to the relatively high moisture content (caused by tempering for 45 min), and the protease at the pH optimum of 4.4 was more accessible to microwaves. However, the reduction in the decrease ratio for the activity of acidic protease (pH 4.4) suggested the synthesis of heat-tolerant proteinase with longer sprouting times.

Therefore, changes in protease activity that occurred in the sprouted wheat with the increase in microwave treatment times (108, 150 and 192 s) were further investigated (Table 2). There was a marked increase in the surface temperature of wheat kernels (from 65 to 85 °C) with increase in microwave heating time. Protease activity decreased significantly (*p* < 0.05) with increase in microwave treatment time. At a microwave treatment time of 108 s (the surface temperature of sprouted wheat kernels was 65 °C), no changes were observed in the activity of neutral protease, and 60–80% of the original acidic protease activities were retained. In contrast, Fahmy [24] reported that the cysteine protease extracted from germinated wheat lost 85% of its activity at 60 ± 4 °C. Possibly, the limited water activity in sprouted wheat kernels restricted the thermal effect to some degree. Specially, the protease activity of both types reached the lowest value of 0 cu/g after the sprouted wheat underwent microwave treatment for 192 s, showing remarkable inactivation effectiveness of the protease on account of the higher microwave energy input. It can be speculated that the synthesis of heat-tolerant protease during wheat germination rendered it tolerant to microwave treatment at 65 °C but sensitive to it at 75 and 85 °C.

### 3.2. Effect of Microwave Treatment on Dough Stretching Characteristics, Dough Mixing Properties and Gluten Quality of Sprouted Wheat

**Figure 1:**
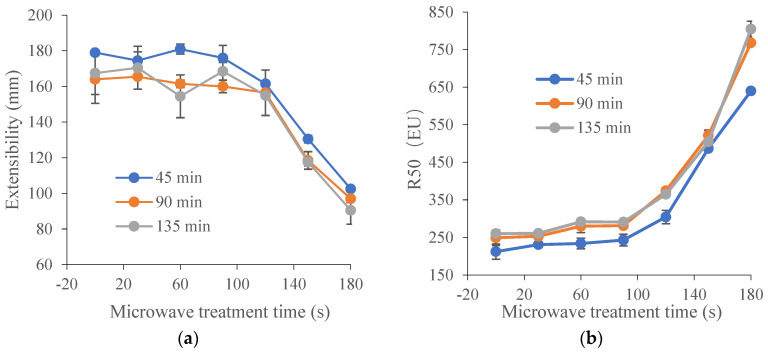

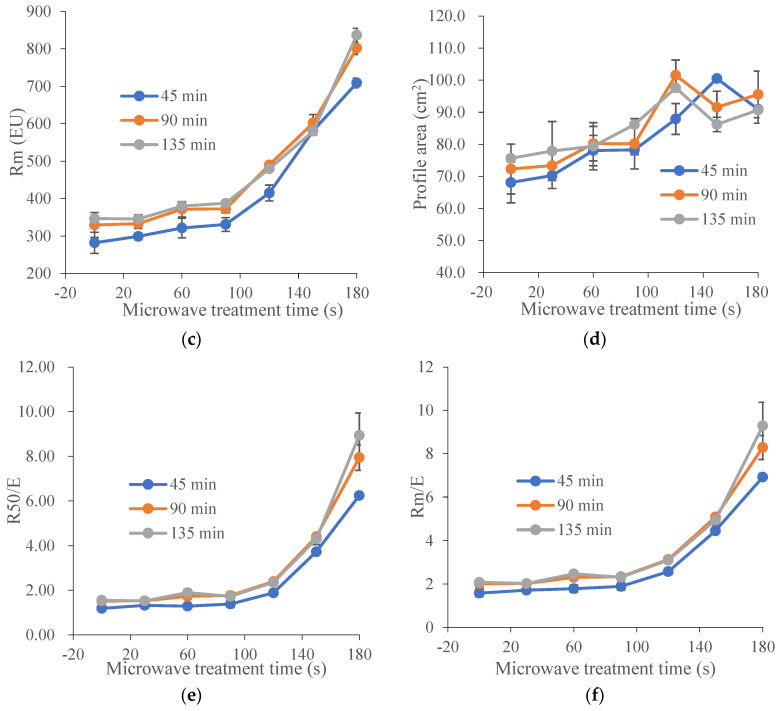
Effect of microwave treatment on the dough extensographic properties of TK6# sprouted for 8 h (TK6#, 8 h). (**a**) Extensibility (E). (**b**) Resistance to constant deformation after 50 mm stretching (R50). (**c**) Maximum of resistance to extension (Rm). (**d**) Profile areas (A, the energy applied for stretching the dough, in cm^2^). (**e**) Ratio between resistance and extensibility (R50/E). (**f**) Maximum of ratio between resistance and extensibility (Rm/E). The tempering time before microwave treatment was 45 min. The microwave treatment times were 0, 30, 60, 90, 120, 150 and 180 s.

Extensograph measurements provided useful information about the viscoelastic behavior of dough. Generally, the extensograph experiments required large amounts of raw materials and a long test time. In order to reduce energy consumption, one variety of wheat (TK6#) was selected. Furthermore, in Section 3.1, it could be concluded that the bulk of the proteolytic activity (approximately 90%) during wheat germination was attribute to the acidic protease (pH 4.4). The decrease ratio for acidic protease activity in TK6#, which sprouted for 8 h, was the highest. Therefore, TK6#, which sprouted for 8 h, was selected as the raw material for the tensile experiments. In our pre-experiment, a longer microwave treatment time, for example, 210 s, caused the wheat kernel to have a slightly burnt taste. Therefore, the microwave treatment times were selected as 0, 30, 60, 90, 120, 150 and 180 s. The effects of microwave treatment on the extensogragh characteristics of the sprouted wheat flour dough samples are presented in Figure 1. The dough extensibility (E), an indicator of the dough processing characteristics, decreased after microwave treatment. Dough made from sprouted wheat was too extensible for optimal baking quality due to protein breakdown and gluten softening. The reduction in dough extensibility compared with untreated sprouted wheat demonstrated the enhancement of dough strength and elasticity. The resistance to extension (parameters R50 and Rm) predicted the dough handling properties and the fermentation tolerance [48]. As a result, the remarkable increase in the values for Rm and R50 induced by microwave treatment suggested an enhanced handling behavior during mechanical mixing and improved tolerance in the fermentation stage. Furthermore, baking performance was related to the interplay between Rm and extensibility, since both factors characterized the extent of the gluten expansion that occurred during the expansion of the gas bubbles. After microwave heating, the bubble walls might have become thinner as the resistance to extension increased, providing bubbles with a greater stability against early coalescence and better gas retention during proofing and baking. Moreover, the energy required for handling the dough (A) and the ratio between resistance and extensibility (R50/E and Rm/E) also increased after microwave treatment. These results showed that microwave treatment led to enhancement of dough strength and improvement of dough elasticity [49] and stability [50]. The decrease in the extensibility and the increase in the resistance to extension of the dough can be attributed to the newly formed disulfide bonds [48], which depended on the absorbed microwave energy, which further contributed to the solidification of the gluten and the increase in dough elasticity. 

As shown in Table 1, the decrease ratio for acidic protease activity in TK6# (sprouted for 8 h and 12 h) and ZM366 (sprouted for 12 h and 16 h), was higher than for the other samples. Therefore, they were selected to investigate the effect of microwave treatment time on the protease activity in sprouted wheat. As shown in Figure 1, the dough strength and elasticity were significantly enhanced when the microwave treatment time exceeded 100 s. In our pre-experiment, a longer microwave treatment time, for example, 210 s, caused the wheat kernels to have a slightly burnt taste. Therefore, microwave treatment times of 0, 108, 150 and 192 s were selected for the following analysis. 

Microwave heating brought about some significant changes in the dough mixing behavior of the sprouted wheat as measured by the Mixolab (Table 3). Gluten played a key role in determining the unique baking quality of wheat by conferring water-absorption capacity, cohesivity, viscosity and elasticity on the dough [11]. The first stage in a Mixolab curve represents the protein properties of dough when subjected to a dual mechanical shear stress, expressed as water absorption, C2 and stability [51]. The water absorption decreased gradually with the increasing time of microwave treatment, suggesting weakening in the water-absorbing capacity of the dough. Microwave heating caused protein unfolding, and more hydrophobic moieties in the protein were exposed, leading to low water-absorption ability. C2 and stability were indicators of gluten quality and dough strength [52], with higher values indicating stronger dough. A significant increase in stability and C2 was observed after microwave treatment, indicating the enhancement of dough resistance and strength [53]. During dough mixing, the water hydrated the flour components and the dough developed. Variation in the hydration behavior of gluten protein may be the reason for differences in dough mixing characteristics. As was shown in Table 3, the gluten index (GI) increased while the wet gluten (WG) content decreased as the level of microwave energy increased. A similar study was reported by Kaasova [8]. The decrease in wet gluten content could probably be explained by the lower water-binding and -holding capacity of the microwave-heated gluten under the conditions of standard washing, which is further supported by the decreased water absorption (WA) of the dough (Table 3). The gluten index (GI) is a characteristic connected with the extensibility and rigidity of wet gluten. Due to heat denaturation, gluten, with a lower water-binding capacity, was probably made less rigid and less extensible, leading to the enhancement of dough strength and the weakening of dough extensibility (Figure 1).

### 3.3. Effect of Microwave Treatment on Protein Solubility

There is general agreement about the link between heating and protein cross-linking. Therefore, studies were conducted on protein solubility, which would serve as an indicator for the degree of cross-linking occurring during microwave heating. Typically, wheat proteins are classified into albumin/globulin, gliadin and glutenin fractions by sequential extraction in dilute salt solutions, aqueous ethanol and dilute acid [42]. As shown in Table 4, the solubility of salt-soluble protein (SSP) and alcohol-soluble protein (ASP) decreased as the microwave treatment time increased at all sprouting levels for both wheat cultivars. These results were in accordance with those of Erkan [54], who reported that the solubility of microwave-heated gluten proteins gradually decreased as the treatment time increased. The contents of acetic acid-insoluble gluteinins (AAIGs) gradually increased, indicating increased protein polymerization. As previously reported, AAIGs were highly correlated with the rheological properties of dough and the breadmaking quality of end-use products [55]. The unextractable polymeric protein fractions could enhance dough strength. These results correspond to the enhancement of dough strength and elasticity described in Section 3.2.

Protein solubility loss was assumed to derive from the increasing connectivity of gluten protein. Microwave heating caused protein unfolding, resulting in the exposition of thiol groups. Protein aggregation might occur between exposed groups through disulfide bond cross-linking, consequently leading to a decrease in protein solubility. 

### 3.4. Analysis of SE-HPLC (Size-Exclusion High-Performance Liquid Chromatography)

Generally, variation in protein solubility is attributed to changes in the molecular weight of proteins. SE-HPLC is an excellent indirect method for characterizing the molecular size distribution of polymeric proteins of wheat, and protein extractability is a good indication of the degree of crosslinking. Wheat endosperm storage proteins consist of two major groups of proteins, monomeric gliadins and polymeric glutenins [11], both of which play a key role in determining the unique baking quality of wheat by conferring water-absorption capacity, cohesivity, viscosity and elasticity on dough. As shown in Table 5, the peak areas of glutenin, gliadin and gluten in sprouted wheat decreased significantly with the increase in microwave energy in the absence or presence of sonication. These phenomena corresponded to the increase in the content of acetic acid-insoluble glutenins (AAIGs) (Table 4) and can be explained by the formation of covalent cross-links induced by microwave heating [29]. Microwave heating of gluten resulted in large protein aggregates which could not be extracted by 50% 1-propanol aqueous solution. Furthermore, the percentage of decreased soluble glutenin was much higher than that of gliadin, suggesting more cross-linking related to glutenin in sprouted wheat after microwave treatment. This can be ascribed to the fact that glutenin was more susceptible to heat treatment than gliadin. 

In previous studies, it was generally accepted that when glutenins were heated above 55 °C or gliadins above 70 °C, sulfhydryl (SH) oxidation reactions occurred mainly [29]. At higher temperatures (up to 90 °C), gliadin links to glutenin through a sulfhydryl–disulfide exchange mechanism [31]. The glutenins have been reported to be extremely important in imparting strength and elasticity to dough [56], while the gliadins confer viscous properties [57]. This confirms that the increased cross-links of glutenin contributed to the improvement of gluten quality (Table 3) and the enhancement of dough strength (Figure 1). All these positive effects can be explained by the heat-triggered protease inactivation (especially of cysteine proteinases) which lessened the cleavage of disulfide bonds in glutenin, simultaneously coupled with the heat-induced covalent cross-linking of gluten during the microwave treatment. Microwave heating led to a slight decrease in the extractability of gliadin, which was beneficial for the viscosity rise. However, the gliadin extractability loss was less pronounced due to the lower wheat temperature (65, 75 and 85 °C in Table 2) after microwave treatment. In previous studies, it was proposed that cross-links among gliadins were formed above 70 °C with glutenins [32,58]. It was of note that cysteine proteinases can cleave both intramolecular and intermolecular disulfide (SS) bonds. In a similar way, the disulfide bonds can be rebuilt in or between gluten proteins due to the combined effectiveness of the inactivation of cysteine proteinases and microwave heating. 

Compared with simple continuous shaking, the protein solubility of sprouted wheat increased after sonication, corresponding to the study of Singh [59]. In particular, as extractable gluten increased after sonication, the proportions of the glutenin fractions increased, coincident with the previous study of Morel [44], who found that the proportions of the earliest eluted SE-HPLC fractions (glutenin) increased after sonication. Generally, the viscoelastic three-dimensional network is formed and stabilized by covalent bonds and superimposed by non-covalent interactions, such as hydrogen bonds, ionic bonds and hydrophobic bonds [60]. Possibly, due to its cavitation effect, sonication could alter the intermolecular non-covalent bonding that is responsible for holding the protein chains together. The protein in sprouted wheat was successfully extracted by sonication instead of chemical reduction through breakdown of large SDS-insoluble glutenin polymers into smaller SDS-soluble polymers.

### 3.5. Analysis of SDS-PAGE (Sodium Dodecyl Sulfate–Polyacrylamide Gel Electrophoresis)

**Figure 2:**
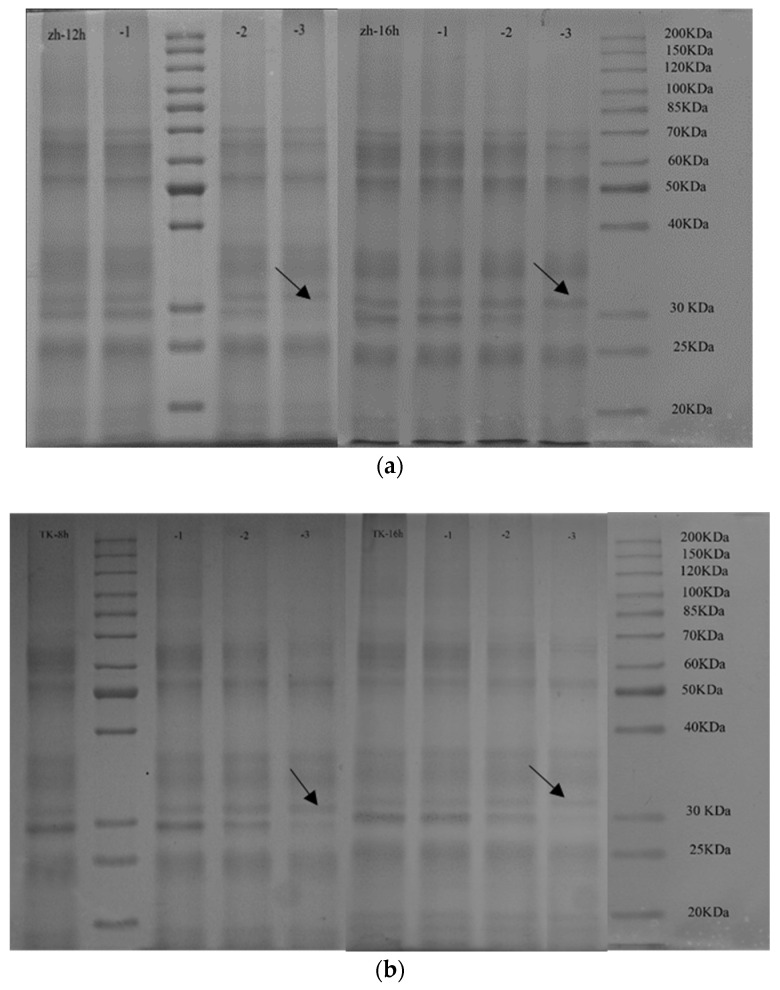

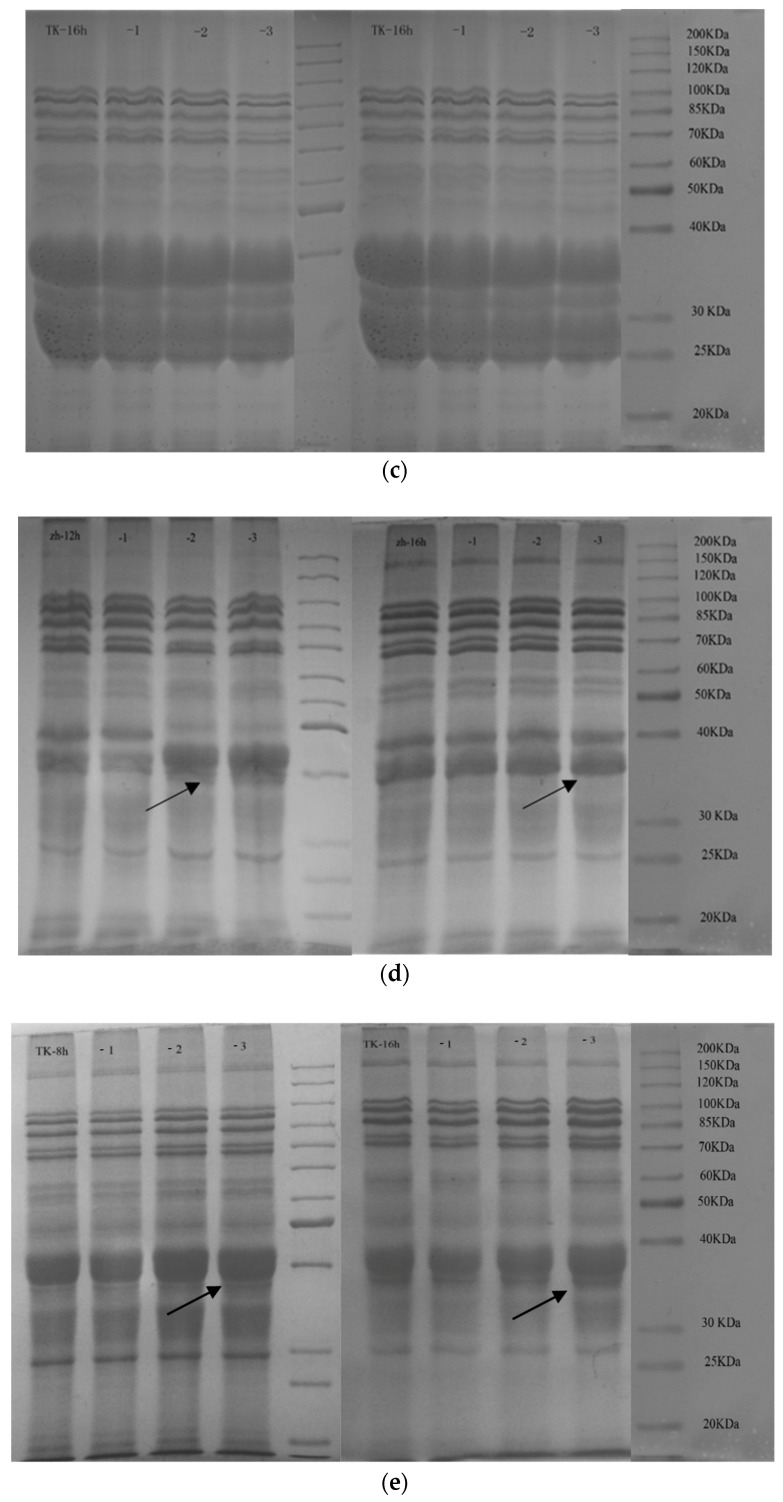
Effect of microwave treatment on the electrophoresis profiles of protein in sprouted wheat after microwave treatment. (**a**) Salt-soluble protein of zh (ZM366) sprouted for 12 and 16 h. (**b**) Salt-soluble protein of TK6# sprouted for 8 and 16 h. (**c**) Alcohol-soluble gliadin of zh (ZM366) and TK6#. (**d**) Unextractable glutenin of zh (ZM366) sprouted for 12 and 16 h. (**e**) Unextractable glutenin of TK6# sprouted for 8 and 16 h. −1, −2, and −3, microwave treatment for 108, 150 and 192 s. The tempering time before microwave treatment was 45 min.

To further evaluate protein changes in molecular size distribution, non-reduced (Figure 2a–c) and reduced (Figure 2d,e) SDS-PAGE was performed. As shown in Figure 2, in the range of approximately 65 kD to 30 kD, the intensity of salt-soluble protein (SSP) in ZM366 (Figure 2a) and TK6# (Figure 2b) showed a slight but obvious decrease with increased microwave heating time, in accordance with the reduction in SSP solubility (Table 4). This could be explained by an aggregation of the molecules into high-molecular-weight units which were no longer soluble. It can be inferred that salt-soluble proteins (albumins and globulins) with a molecular weight range of approximately 65 kD to 30 kD were involved in the protein cross-linking to a lesser extent.

More specifically, evidence for disulfide bond cross-linking in glutenin was obtained by separating the reduced glutenin fractions in SDS-PAGE profiles (Figure 2d,e). Glutenin subunits consisted of high-molecular-weight (HMW-GSs) and low-molecular-weight glutenin subunits (LMW-GSs). A slight increase in the band intensity was observed in high-molecular-weight glutenin subunits (HMW-GSs) of 85.0–100.0 KDa (x-HMW-GSs) and 60–70 KDa (y-HMW-GSs), indicating that an increase in disulfide bond cross-linking is involved in HMW-GSs. The LMW-GSs, which were classified as B, C and D types, could form both intrachain and interchain disulfide bonds among themselves and with HMW-GSs [11]. The polymerization seemed to mainly involve B-type and C-type low-molecular-weight glutenin subunits (B/C-LMW-GSs) [61], as evidenced by the obvious increase in the band intensity in the range of 30–50 kD, especially in wheat with a lower sprouting degree (ZM366 sprouted for 12 h and TK6# sprouted for 8 h). This corresponded to the increased contents of acetic acid-insoluble gluteinins (AAIGs) shown in Table 4 and the decrease in extractable glutenin shown in Table 5. The LMW-GSs are reported to be the major determinants of the viscoelasticity of wheat gluten [62]. More cross-linking related to LMW-GSs possibly improved the technological quality of sprouted wheat dough. It could be concluded that the increased cross-linking related to glutenins, including HMW-GSs and LMW-GSs, contributed mostly to the enhancement of gluten indexes (Table 3) and dough strength (Figure 1), enabling the capacity to retain gas and maintain great resilience to cope with starch swelling during product processing.

With respect to alcohol-soluble protein (ASP), no significant differences were detected in the profiles of SDS-PAGE due to the lower microwave treatment temperatures. These results were in line with the results presented in Figure 2 and Table 5. According to a previous report, the denaturation temperature of gliadin was higher than that of glutenin [63]. A minimum temperature of 55 °C was necessary to polymerize glutenins, while gliadins needed even higher temperatures to copolymerize with glutenins. Traditionally, gliadins are conceptualized as monomeric proteins that form only intramolecular disulfide bonds, if present, whereas glutenins are polymeric proteins whose subunits are held together by intermolecular disulfide bonds, although intrachain bonds are also present [63]. Therefore, glutenin’s inner structure was more easily exposed than that of gliadin in the case of microwave heating, and, consequently, more cross-links were formed in glutenin than in gliadin.

Possibly, microwave heating first resulted in conformational changes in gluten, exposing previously unavailable areas (possibly containing free SH groups), after which polymerization and cross-linking of glutenin via disulfide bonds with oxidation of SH-groups occurred due to microwave treatment. The absence of free SH groups and a lower temperature held back the exposure of reactive amino acid side chains in gliadin, preventing gliadin from being incorporated in the protein network.

### 3.6. Analysis of RP-HPLC (Reversed-Phase High-Performance Liquid Chromatography)

**Figure 3:**
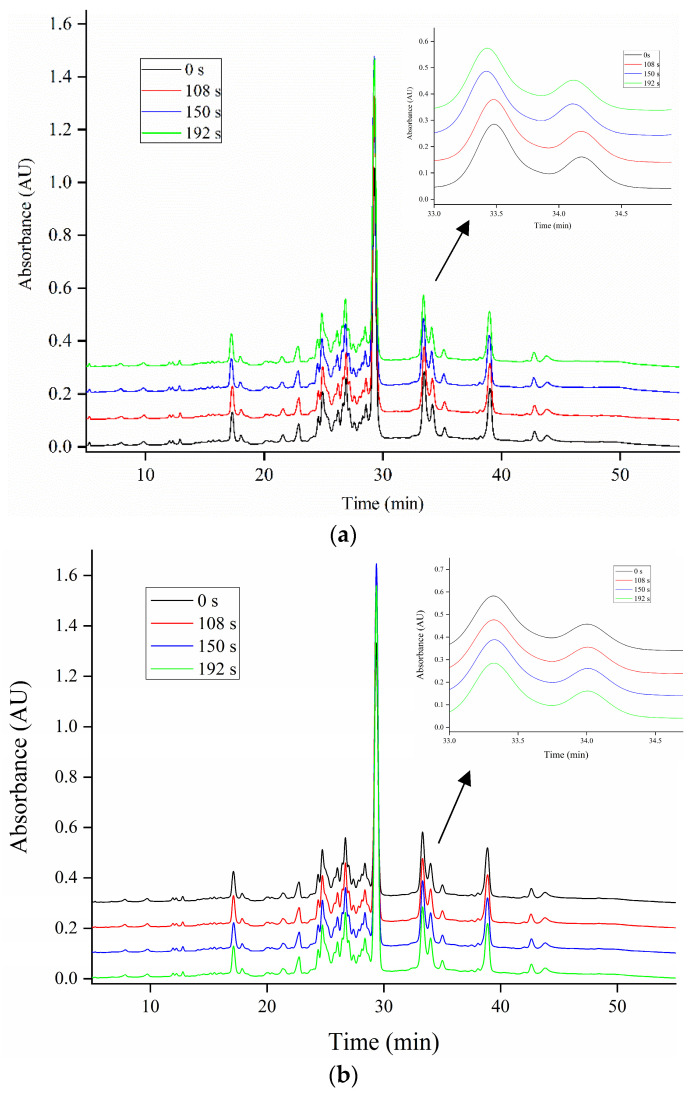
Effect of microwave treatment on the RP-HPLC profiles of gliadin subunits in sprouted wheat. (**a**) ZM366 sprouted for 12 h. (**b**) ZM366 sprouted for 16 h. The tempering time before microwave treatment was 45 min.

**Figure 4:**
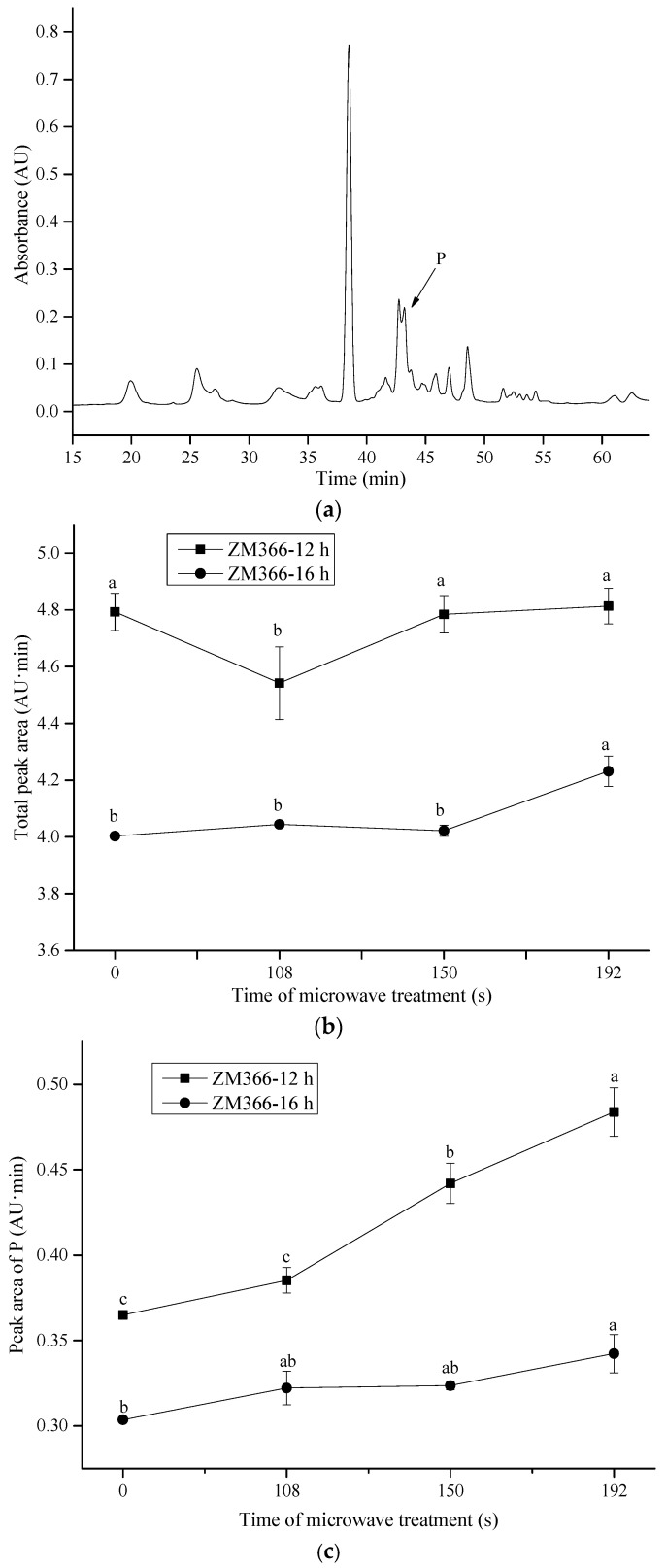
Effect of microwave treatment on insoluble glutenin subunits in sprouted wheat. (**a**) The RP-HPLC profile of glutenin in sprouted wheat. (**b**) The total peak area of glutenin subunits. (**c**) The peak area of P fractions in glutenin. The tempering time before microwave treatment was 45 min. Different letters indicate significant differences (*p* < 0.05).

To further elaborate on the relative contribution of the gluten subunits to the cross-linking, RP-HPLC profiles of gliadin and glutenin subunits in ZM366 are shown in Figure 3 and Figure 4. As the microwave treatment progressed, no significant changes in the levels of gliadin subunits (α-, γ- and ω-gliadins) were observed (Figure 3). This could be explained by the fact that all cysteine residues in α- and γ-gliadins were involved in intrachain disulfide bonds, while ω-gliadins lack cysteine residues. None of them was reactive at lower temperatures (<90 °C) [64]. Slight differences in RP-HPLC profiles existed for total insoluble glutenin subunits (Figure 4b) that had been submitted to microwave treatment. In particular, a significant increase in areas of P fractions (Figure 4a,c) was observed after microwave treatment, coinciding with the increased intensity of LMW-GSs in SDS-PAGE (Figure 2d,e). The above observation verified that cross-linking related to LMW-GSs, as mentioned above, contributed most to the polymerization and cross-linking of gluten which were induced by microwave heating. Generally, LMW-GSs are associated with dough resistance and extensibility [11,65]. Cross-linking involving LMW-GSs might have contributed to the deformation resistance and balance of dough strength and extensibility after microwave treatment, which corresponded to the enhancement of dough properties described in Section 3.2.

## 4. Conclusions

In this study, protease inactivation induced by microwave heating hindered protein hydrolysis, alleviating protein weakening and enhancing dough strength. Furthermore, protein cross-linking induced by the microwave heating contributed mostly to the enhancement of gluten indexes and dough strength. Therefore, it could be concluded that microwave treatment led to significant (*p* < 0.05) improvement in the dough properties and gluten quality of sprouted wheat due to the synergistic effectiveness of protease inactiveness and protein cross-linking. Microwave treatment led to a significant (*p* < 0.05) reduction in protease activity in sprouted wheat, and proteases with an optimum pH of 4.4 (cysteine proteinases) were more susceptible to microwave heating, contributing most to the protease inactiveness. The heat-induced disulfide bond cross-linking of gluten that occurred seemed to mainly involve glutenin, especially the B/C LMW-GSs (30–50 kD), whereas the changes in gliadin were less pronounced due to the lower temperatures (<90 °C) used for microwave treatment. At present, microwave treatment of sprouted wheat has entered the pilot stage and efforts will be made to promote its industrial application.

## Figures and Tables

**Table 1:** Effects of microwave treatment on protease activity in sprouted wheat with different sprouting degrees.

	pH 7.5						pH 4.4					
ST	TK6# (u/g)		DR	ZM366 (u/g)		DR	TK6# (u/g)		DR	ZM366 (u/g)		DR
(h)	SW ^A^	SWMT ^B^	(%)	SW ^A^	SWMT ^B^	(%)	SW ^A^	SWMT ^B^	(%)	SW ^A^	SWMT ^B^	(%)
0	10.40 ± 4.01 d	-	-	3.78 ± 1.78 c	-	-	199.40 ± 1.34 g	-	-	190.89 ± 0.89 f	-	-
8	13.23 ± 2.67 d	6.62 ± 1.34 b	50.00	-	-	-	296.73 ± 0.00 f	40.64 ± 1.34 e	86.30	-	-	-
10	-	-	-	7.56 ± 1.78 c	0 ± 0 b	100	-	-	-	389.97 ± 2.67 e	-	-
12	16.07 ± 1.34 cd	6.62 ± 1.34 b	58.81	8.19 ± 2.67 c	0 ± 0 b	100	348.71 ± 4.01 e	100.17 ± 5.35 d	71.27	415.17 ± 0.89 d	42.84 ± 1.78 d	89.68
16	18.90 ± 2.67 cd	8.51 ± 1.34 b	54.97	14.49 ± 0.89 b	8.82 ± 1.78 a	39.13	378.95 ± 1.34 d	193.73 ± 1.34 c	48.88	437.22 ± 5.35 c	54.18 ± 1.78 c	87.61
20	23.63 ± 6.68 bc	10.40 ± 4.01 ab	55.99	15.75 ± 0.89 ab	9.45 ± 0.89 a	40.00	440.37 ± 0.00 c	-	-	456.12 ± 1.78 b	124.11 ± 6.24 b	72.79
24	30.24 ± 2.67 ab	14.18 ± 1.34 a	53.11	19.53 ± 2.67 a	10.08 ± 0.00 a	44.70	477.23 ± 4.01 b	232.47 ± 8.02 b	51.29	535.51 ± 3.56 a	363.51 ± 0.89 a	32.12
48	35.91 ± 2.67 a	15.15 ± 0.00 a	57.81	-	-	-	493.29 ± 5.35 a	433.76 ± 1.34 a	12.07	-	-	-

Capitals in superscript in the same row mean that protease activity in SW is significantly different (*p* < 0.05) from that in SWMT. Means followed by different letters in the same column are significantly different (*p* < 0.05). ST, sprouting time; SW, sprouted wheat; SWMT, sprouted wheat after microwave treatment; DR, decrease ratio; -, not detected. The tempering time was 45 min, and the microwave treatment time was 150 s.

**Table 2:** Effects of microwave treatment time on the protease activity in sprouted wheat.

		pH 7.5				pH 4.4			
MTT	STWK	TK6#, 8 h	TK6#, 16 h	ZM366, 12 h	ZM366, 16 h	TK6#, 8 h	TK6#, 16 h	ZM366, 12 h	ZM366, 16 h
(s)	(°C)	(u/g)	(u/g)	(u/g)	(u/g)	(u/g)	(u/g)	(u/g)	(u/g)
0	-	13.23 ± 2.67 a	18.90 ± 2.67 a	8.19 ± 2.67 a	14.49 ± 0.89 a	296.73 ± 0.00 a	378.95 ± 1.34 a	415.17 ± 0.89 a	437.22 ± 5.35 a
108	65 c	13.23 ± 0.00 a	17.96 ± 1.34 a	8.19 ± 0.89 a	14.49 ± 2.67 a	243.81 ± 2.67 b	286.34 ± 1.34 b	301.77 ± 0.89 b	268.38 ± 1.78 b
150	75 b	7.56 ± 2.67 b	8.5 ± 1.34 b	0 ± 0 b	10.08 ± 1.78 a	18.90 ± 2.67 c	106.79 ± 6.68 c	44.73 ± 0.89 c	112.14 ± 0.00 c
192	85 a	0 ± 0 c	0 ± 0 c	0 ± 0 b	0 ± 0 b	0 ± 0 d	0 ± 0 d	0 ± 0 d	0 ± 0 d

MTT, microwave treatment time; STWK, surface temperature of wheat kernels; TK6#, 8 h, 16 h, TK6# sprouting for 8 h and 16 h; ZM366, 12 h, 16 h, ZM366 sprouting for 12 h and 16 h. Means followed by different letters in the same column are significantly different (*p* < 0.05). The tempering time before microwave treatment was 45 min.

**Table 3:** Effects of microwave treatment on the Mixolab parameters and gluten quality of sprouted wheat flour.

Samples	MTT	WA	Stability	C2	WG	GI
	(s)	(%)	(min)	(Nm)	(%)	(%)
TK6#, 8 h	0	51.20 ± 0.14 a	3.99 ± 0.09 c	0.33 ± 0.00 d	29.00 ± 0.14 a	54.00 ± 1.41 c
	108	50.80 ± 0.00 b	5.35 ± 0.45 b	0.36 ± 0.01 c	28.66 ± 0.07 a	53.50 ± 0.71 c
	150	50.10 ± 0.00 c	8.69 ± 0.44 a	0.41 ± 0.01 b	25.57 ± 0.00 b	74.50 ± 0.71 b
	192	50.05 ± 0.07 c	4.70 ± 0.61 bc	0.46 ± 0.01 a	19.00 ± 0.28 c	98.50 ± 0.71 a
TK6#, 16 h	0	51.85 ± 0.07 a	3.58 ± 0.08 b	0.28 ± 0.00 c	28.90 ± 0.14 a	43.50 ± 0.71 c
	108	50.50 ± 0.00 b	3.77 ± 0.23 b	0.29 ± 0.00 c	28.90 ± 0.14 a	44.50 ± 0.71 c
	150	50.45 ± 0.07 b	6.13 ± 0.21 a	0.34 ± 0.01 b	25.53 ± 0.14 b	62.5 ± 2.12 b
	192	50.05 ± 0.07 c	3.34 ± 0.45 b	0.39 ± 0.01 a	18.50 ± 0.14 c	89.50 ± 0.71 a
ZM366, 12 h	0	61.70 ± 0.57 a	7.26 ± 0.32 d	0.34 ± 0.01 c	32.61 ± 0.29 a	84.45 ± 4.97 c
	108	59.60 ± 0.00 b	8.56 ± 0.08 c	0.41 ± 0.00 b	32.10 ± 0.07 a	91.21 ± 0.91 b
	150	58.75 ± 0.64 bc	10.06 ± 0.13 b	0.45 ± 0.03 b	28.80 ± 0.36 b	98.78 ± 0.23 a
	192	57.60 ± 0.00 c	10.88 ± 0.18 a	0.54 ± 0.00 a	22.30 ± 0.14 c	100.00 ± 0.00 a
ZM366, 16 h	0	60.90 ± 0.00 a	5.79 ± 0.02 d	0.28 ± 0.01 c	33.23 ± 0.00 a	90.40 ± 3.66 c
	108	59.00 ± 0.00 b	7.09 ± 0.05 b	0.31 ± 0.02 c	32.33 ± 0.29 b	92.81 ± 0.38 bc
	150	58.30 ± 0.14 c	9.54 ± 0.05 a	0.35 ± 0.01 b	29.22 ± 0.36 c	97.07 ± 1.19 ab
	192	58.10 ± 0.00 d	6.02 ± 0.10 c	0.41 ± 0.01 a	22.15 ± 0.07 d	100.00 ± 0.00 a

WA, water absorption; WG, wet gluten; GI, gluten index; Stability, the duration for which the Mixolab curve remains within 11% of the maximum consistency obtained during the mixing phase; C2, the minimum value of dough consistency, the combined effect of heating and mechanical shear stress (mixing) led to unfolding and destabilization of proteins, inducing a decrease in dough consistency to C2 torque that measured protein weakening. Means followed by different letters in the same column are significantly different (*p* < 0.05).

**Table 4:** Effects of microwave treatment on protein solubility in sprouted wheat.

Samples	MTT	SSP	ASP	AASG	AAIG
	(s)	(%)	(%)	(%)	(%)
TK6#, 8 h	0	23.14 ± 0.03 b	36.63 ± 0.15 a	6.75 ± 0.19 b	33.49 ± 0.14 d
	108	23.66 ± 0.05 a	35.56 ± 0.11 b	4.03 ± 0.05 d	36.75 ± 0.12 c
	150	23.69 ± 0.15 a	33.24 ± 0.33 c	5.45 ± 0.16 c	37.61 ± 0.01 b
	192	19.93 ± 0.01 c	30.89 ± 0.01 d	7.64 ± 0.16 a	41.54 ± 0.17 a
TK6#, 16 h	0	23.16 ± 0.16 a	37.23 ± 0.06 a	6.71 ± 0.25 b	32.90 ± 0.12 d
	108	21.95 ± 0.13 b	35.25 ± 0.16 b	7.67 ± 0.22 a	35.13 ± 0.07 c
	150	21.43 ± 0.08 c	33.96 ± 0.01 c	6.53 ± 0.05 b	38.08 ± 0.13 b
	192	19.20 ± 0.22 d	33.46 ± 0.17 d	7.75 ± 0.31 a	39.60 ± 0.19 a
ZM366, 12 h	0	23.46 ± 0.18 b	36.20 ± 0.11 b	6.42 ± 0.22 a	33.92 ± 0.33 d
	108	24.53 ± 0.26 a	34.37 ± 0.45 d	5.86 ± 0.24 b	35.24 ± 0.17 c
	150	21.03 ± 0.05 c	35.56 ± 0.98 c	5.09 ± 0.26 c	38.31 ± 0.76 b
	192	19.25 ± 0.11 d	36.55 ± 0.17 a	5.09 ± 0.35 c	39.11 ± 0.15 a
ZM366, 16 h	0	23.56 ± 0.10 a	36.26 ± 0.14 a	6.46 ± 0.33 ab	33.72 ± 0.23 c
	108	20.08 ± 0.18 b	34.76 ± 0.24 b	6.73 ± 0.19 a	38.43 ± 0.11 b
	150	20.09 ± 0.20 b	33.59 ± 0.18 c	6.51 ± 0.24 ab	39.82 ± 0.19 a
	192	19.76 ± 0.27 c	33.90 ± 0.18 c	6.22 ± 0.32 b	40.12 ± 0.32 a

MTT, microwave treatment time; SSP, salt-soluble protein; ASP, alcohol-soluble protein; AASG, acetic acid soluble gluteinin; AAIG, acetic acid insoluble glutenin. Means followed by different letters in the same column are significantly different (*p* < 0.05).

**Table 5:** Effects of microwave treatment on the peak areas of soluble proteins extracted with 50% 1-propanol aqueous solution in SE-HPLC (size-exclusion high-performance liquid chromatography) profiles.

		Traditional Extraction			Ultrasonic-Assisted Extraction		
Samples	MTT	Glutenin	Gliadin	Gluten	Glutenin	Gliadin	Gluten
	(s)	(AU·min)	(AU·min)	(AU·min)	(AU·min)	(AU·min)	(AU·min)
TK6#, 8 h	0	0.6830 ± 0.0014 a	1.5652 ± 0.0008 a	2.2482 ± 0.0022 a	1.0195 ± 0.0089 a	1.7392 ± 0.0033 a	2.7587 ± 0.0057 a
	108	0.6425 ± 0.0001 b	1.5420 ± 0.0038 b	2.1845 ± 0.0038 b	0.9378 ± 0.0167 b	1.6806 ± 0.0382 b	2.6184 ± 0.0549 b
	150	0.5447 ± 0.0003 c	1.5146 ± 0.0009 c	2.0593 ± 0.0012 c	0.8804 ± 0.0043 c	1.6798 ± 0.0156 b	2.5601 ± 0.0199 bc
	192	0.4326 ± 0.0020 d	1.4466 ± 0.0020 d	1.8792 ± 0.0040 d	0.8521 ± 0.0059 d	1.6697 ± 0.0001 b	2.5218 ± 0.0059 c
TK6#, 16 h	0	0.7069 ± 0.0063 a	1.5103 ± 0.0046 a	2.2172 ± 0.0016 a	1.0822 ± 0.0238 a	1.7134 ± 0.0156 a	2.7956 ± 0.0394 a
	108	0.6914 ± 0.0031 b	1.5039 ± 0.0066 a	2.1953 ± 0.0097 b	1.0618 ± 0.0124 a	1.6910 ± 0.0090 a	2.7527 ± 0.0214 a
	150	0.5846 ± 0.0015 c	1.4812 ± 0.0009 b	2.0658 ± 0.0023 c	0.9556 ± 0.0052 b	1.6500 ± 0.0009 b	2.6056 ± 0.0043 b
	192	0.4573 ± 0.0030 d	1.4113 ± 0.0019 c	1.8686 ± 0.0049 d	0.9267 ± 0.0050 b	1.6515 ± 0.0030 b	2.5781 ± 0.0081 b
ZM366, 12 h	0	0.6441 ± 0.0006 a	1.8249 ± 0.0033 a	2.4690 ± 0.0039 a	1.0908 ± 0.0076 b	1.7134 ± 0.0156 a	2.8042 ± 0.0080 a
	108	0.6254 ± 0.0008 b	1.8108 ± 0.0003 b	2.4362 ± 0.0011 b	1.1519 ± 0.0081 a	1.6910 ± 0.0090 a	2.8429 ± 0.0170 b
	150	0.5565 ± 0.0004 c	1.7941 ± 0.0006 c	2.3506 ± 0.0010 c	1.0542 ± 0.0021 c	1.6950 ± 0.0062 a	2.7492 ± 0.0083 c
	192	0.4719 ± 0.0036 d	1.7376 ± 0.0063 d	2.2095 ± 0.0099 d	0.9411 ± 0.0046 d	1.6515 ± 0.0030 b	2.5925 ± 0.0076 d
ZM366, 16 h	0	0.6596 ± 0.0012 a	1.7994 ± 0.0017 a	2.4590 ± 0.0029 a	1.1888 ± 0.0152 a	1.9616 ± 0.0024 a	3.1504 ± 0.0128 ab
	108	0.6408 ± 0.0002 b	1.7979 ± 0.0003 a	2.4387 ± 0.0005 b	1.1777 ± 0.0186 a	1.9854 ± 0.0021 a	3.1630 ± 0.0399 a
	150	0.5706 ± 0.0012 c	1.7751 ± 0.0052 b	2.3457 ± 0.0064 c	1.1408 ± 0.0030 b	1.9620 ± 0.0069 a	3.1028 ± 0.0100 ab
	192	0.4964 ± 0.0004 d	1.7438 ± 0.0001 c	2.2402 ± 0.0003 d	1.1246 ± 0.0107 b	1.9759 ± 0.0134 a	3.1005 ± 0.0026 b

Means followed by different letters in the same column are significantly different (*p* < 0.05).

## Data Availability

The original contributions presented in the study are included in the article, further inquiries can be directed to the corresponding author.
